# Clinico‐pathological profiling of peripheral ossifying fibroma of the oral cavity

**DOI:** 10.1002/ccr3.4966

**Published:** 2021-10-13

**Authors:** Ashish Shrestha, Shashi Keshwar, Neetu Jain, Toniya Raut, Mehul Rajesh Jaisani, Shiva Lal Sharma

**Affiliations:** ^1^ BP Koirala Institute of Health Sciences Department of Oral Pathology Dharan Nepal; ^2^ BP Koirala Institute of Health Sciences Department of Oral and Maxillofacial Surgery Dharan Nepal; ^3^ BP Koirala Institute of Health Sciences Department of Periodontology and Oral Implantology Dharan Nepal

**Keywords:** Calcification, Histopathology, Peripheral ossifying fibroma

## Abstract

POF represents a separate entity with diverse histological presentation. Considerable overlapping of clinical and histological features are present among different reactive gingival lesions, which warrant a meticulous review for the diagnosis of POF.

## Graphical abstract

Peripheral ossifying fibroma represents a separate entity with diverse histological presentation. Considerable overlapping of clinical and histological features is present among different reactive gingival lesions, which warrant a meticulous review for the diagnosis of POF.

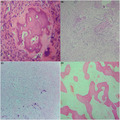

## 1 BACKGROUND

Peripheral ossifying fibroma is a commonly encountered reactive gingival lesion of oral cavity with overlapping clinical and histopathological presentations. Here, we present a series of twenty‐three cases of peripheral ossifying fibroma highlighting epithelial and connective tissue variations, which is significant in critical evaluation of the pathology. Peripheral ossifying fibroma (POF) is a focal, slow growing, reactive lesion arising from pluripotent cells of periodontal ligament, which was first reported by Shepherd as alveolar exostosis in 1844.^1^, ^2^, ^3^, ^4^ The pluripotent cells of the periodontal ligament have apparent ability to transform or metaplastically change into osteoblasts, cementoblasts, or fibroblasts.^5^

Peripheral ossifying fibroma presents as a painless, hemorrhagic, and often lobulated mass of gingiva or alveolar mucosa, with area of surface ulcerations. There is variability in the size of the lesion depending upon amount of superficial inflammation and edema.^5^ POF is histopathologically characterized by the presence of aggregated submucosal proliferation of primitive oval and bipolar mesenchymal cells along with scattered bone, cementum like, or area of dystrophic calcification.^6^, ^7^

The variability of clinical appearance, resemblance to other gingival lesions, and difference in histopathological presentation have created diagnostic dilemma. Here, we present a series of twenty‐three cases of peripheral ossifying fibroma highlighting the variation in clinical presentation and histopathological characteristics.

## 2 CASE DESCRIPTION

### 2.1 Demographic and clinical profile

The present study included 23 diagnosed cases of peripheral ossifying fibroma, which included 16 females (69.56%) and 7 males (30.43%), of age range from 10–70 years, prevalent among 2^nd^, 3^rd^, and 4^th^ decade, respectively. The lesions presented clinically as uni‐focal nodular mass or a swelling present since 1 month–1.5 years, with a case present since 4 years. The size of the lesion ranged from 0.3–3 cm in maximum dimension of which majority were pedunculated (12 cases). Five of the cases were sessile whereas rest of the cases lacks the clinical details to this regard. Anterior region of the jaw was the predominate site (12 cases) of which upper jaw was the most common (10 cases). The cases were heterogeneously associated with all the teeth, that is, incisor, canine, premolars, and molars however central and lateral incisors predominated followed by canine and first premolar with prevalence on right side of the jaw. The lesions were mostly associated with labial and buccal gingiva with five cases, three in palatal and two in lingual gingiva. Two cases in anterior maxilla were extensive to involve both labial and palatal aspect. One case was associated with edentulous ridge in relation to 16.

History of previous excision was noted in four cases of 1 month, 6 months, 1.5 years, and 4 years back. One of the cases among reoccurrence, patient failed to recall the exact duration for reoccurrence. Operative diagnosis of pyogenic granuloma was made for 19 samples (82.6%), one case of irritational fibroma and only three of POF, of which one had a documented histopathological report of peripheral ossifying fibroma on incisional biopsy (Table 1).

**TABLE 1:** Clinical and histopathological profile of diagnosed cases of Peripheral Ossifying Fibroma

Case No.	Age (years)/Sex	Site	Histopathological findings		
			Epithelial ulceration	Types of calcification	Inflammation
1.	10/M	Ant. Max.	‐	I	D
2.	18/M	Ant. Max.	‐	I,II	D
3.	19/M*	Max. I, C PM	+	I,III	F
4.	19/F*	Mand. C, PM	‐	I,II,III	F
5.	21/F	Ant. Max.	+	I,II	D
6.	22/F	Ant. Max.	‐	I,II,III	D
7.	24/F	Post. Mand.	+	I,II,III	D
8.	24/F	Ant. Max.	+	I,II	D
9.	25/F	Ant. Mand.	‐	I	D
10.	27/M*	Post. Mand.	+	I,II	D
11.	31/F	Post. Max.	+	I	D
12.	31/F	Post. Max.	+	I,II,III	D
13.	32/F	Ant. Mand.	+	I	D
14.	35/F	Max. C, PM	+	I	D
15.	39/F	Mand. C, PM	‐	I	F
16.	40/F	Ant. Max.	+	I,II,III	D
17.	40/M	Post. Mand.	+	I	D
18.	45/F*	Post. Mand.	+	I	D
19.	47/F	Ant. Max.	‐	I,III	F
20.	47/M	Ant. Max.	+	I, III	D
21.	52/M*	Ant. Max.	+	I	D
22.	55/F	Ant. Max.	+	I,II,III	D
23.	70/F	Post. Mand.	‐	I	D

Abbreviations: ‐, absent; +, present; Ant., anterior; D, diffuse; F, focal; I, woven and trabeculae type calcification; II, globules like cementum; III, dystrophic calcification; Mand., mandible; Max., maxilla; Post, posterior.

* Recurrent.

#### 2.1.1 Histopathological profile

The histopathological review was carried out based upon microscopic parameters including epithelial presentation, connective tissue features, inflammation, and characteristics of hard tissue (Table 2).^6^, ^8^

**TABLE 2:** Histopathological parameters and criteria

Histopathological parameters	Criteria
Epithelium	Subdivided in normal, atrophic (thin epithelium with less than five cell layers), hyperplastic (epithelium with more than 15–20 cell layers) or absent (areas of ulceration)
Connective tissue	Type of connective tissue ‐ loose or dense; as well as the presence of fibroblastic proliferation dispersed on the connective tissue.
Inflammation	Distribution of inflammatory infiltrate—classified as mild, when there were focal areas, especially on the sub‐epithelial area, or intense, when it was dispersed and deep on the connective tissue. Type of inflammatory infiltrate—predominantly acute, chronic, or both.
Bone/calcification	Type I: Woven and lamellar bone trabeculae Type II: Cementum‑like formations: Mineralized bodies that appear circumscribed, amorphous, almost acellular, basophilic (but sometimes eosinophilic), and range in size from small to very large globules Type III: Dystrophic calcifications: Granular foci of mineralization, which present as a cluster of very small basophilic granules, tiny globules and small, solid irregular masses.

#### 2.1.2 Epithelium

Overlying epithelium was parakeratinized stratified squamous which varies from complete to partial ulcerations and atrophic to hyperplastic. Ulcerations were completely lacking in eight cases (34.78%). Ulcerated epithelium was covered with fibrinous exudate intermixed with acute inflammatory cells infiltrates, neutrophils. Sub‐epithelial zone showed numerous small‐sized blood vessels with red blood cells (Table 1).

#### 2.1.3 Connective tissue

**FIGURE 1:**
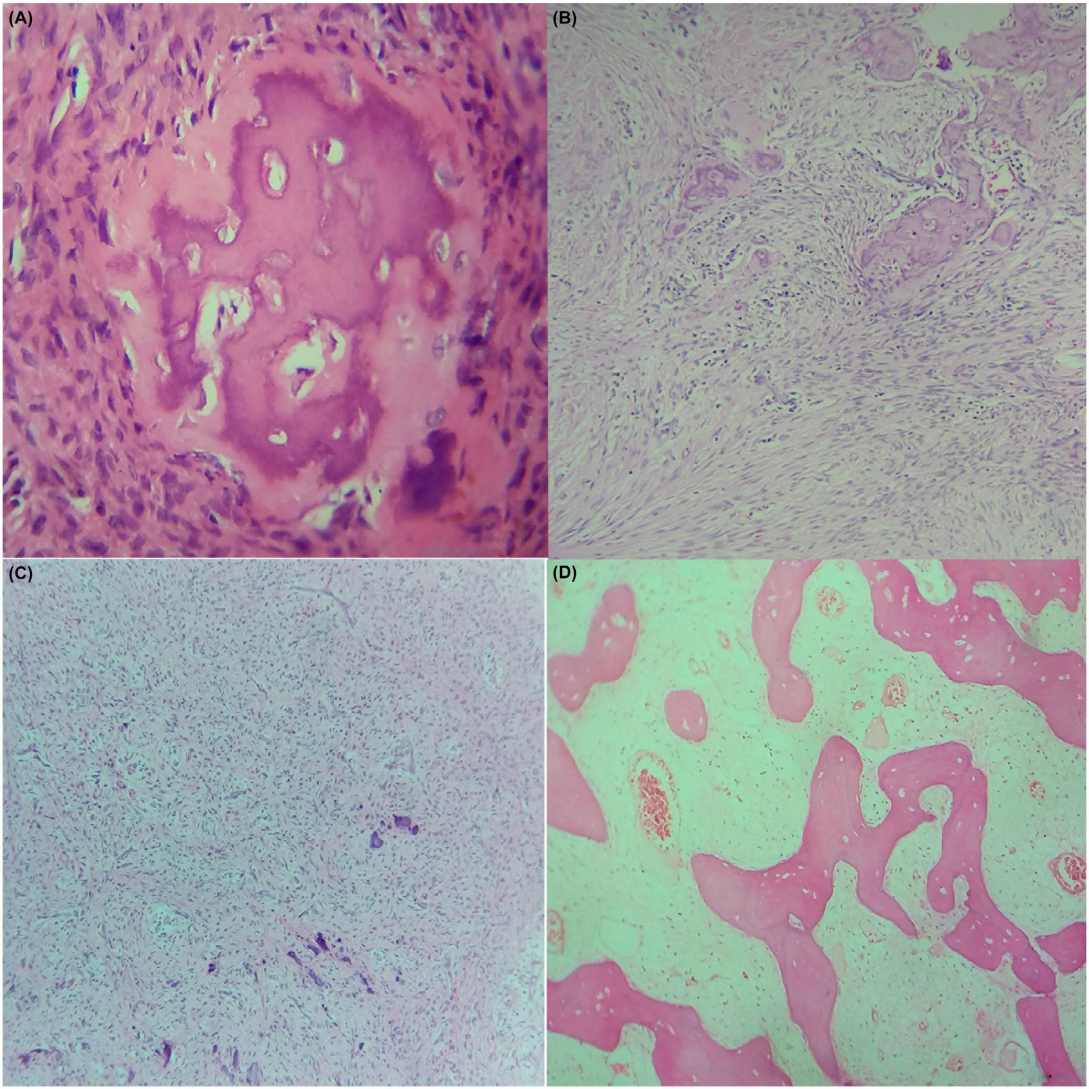
Histopathological features with varying mesenchymal presentations of peripheral ossifying fibroma exhibiting; active fibroblast (A); fasciculate pattern (B); dystrophic calcification in primitive mesenchyme‐like background (C); interconnecting trabecular pattern of lamellar bone with loosely cellular surrounding areas (D; 10×, H&E)

Field‐wise variations were noted within the connective tissue stroma. Few areas were fibrous whereas few were highly cellular. Fibrous component was basically of two patterns, one with thin delicate fibers strands in sparsely cellular background and another with thick collagen fibers arranged in bundles. Thin strands of fibers were mainly seen in cases with complete ulceration. Dense cellularity of the stroma was mainly credited to the active fibroblasts of the stroma (Figure 1A). Fibroblast typically presented as round to oval cells with elongated vesicular nuclei randomly distributed around areas of woven bone formation. Few cases showed fasciculate pattern of these spindle‐shaped fibroblast (Figure 1B). Few cases showed high cellularity resembling primitive mesenchyme‐like feature mainly around area of cementum‐like calcification (Figure 1C). Connective tissue around lamellar trabeculae of bone was comparatively loose type (Figure 1D).

#### 2.1.4 Inflammation

Inflammatory component was both acute and chronic type predominated by neutrophils, lymphocytes, and plasma cells, respectively. Neutrophils were mainly in the superficial layers showing epithelial ulceration admixed with fibrinous exudates; whereas chronic inflammatory cells showed either diffusely distributed throughout the stroma or present in multifocal aggregates. Few cases showed focal aggregation of precisely of plasma cells with Russell body formations.

#### 2.1.5 Bone/calcification

**FIGURE 2:**
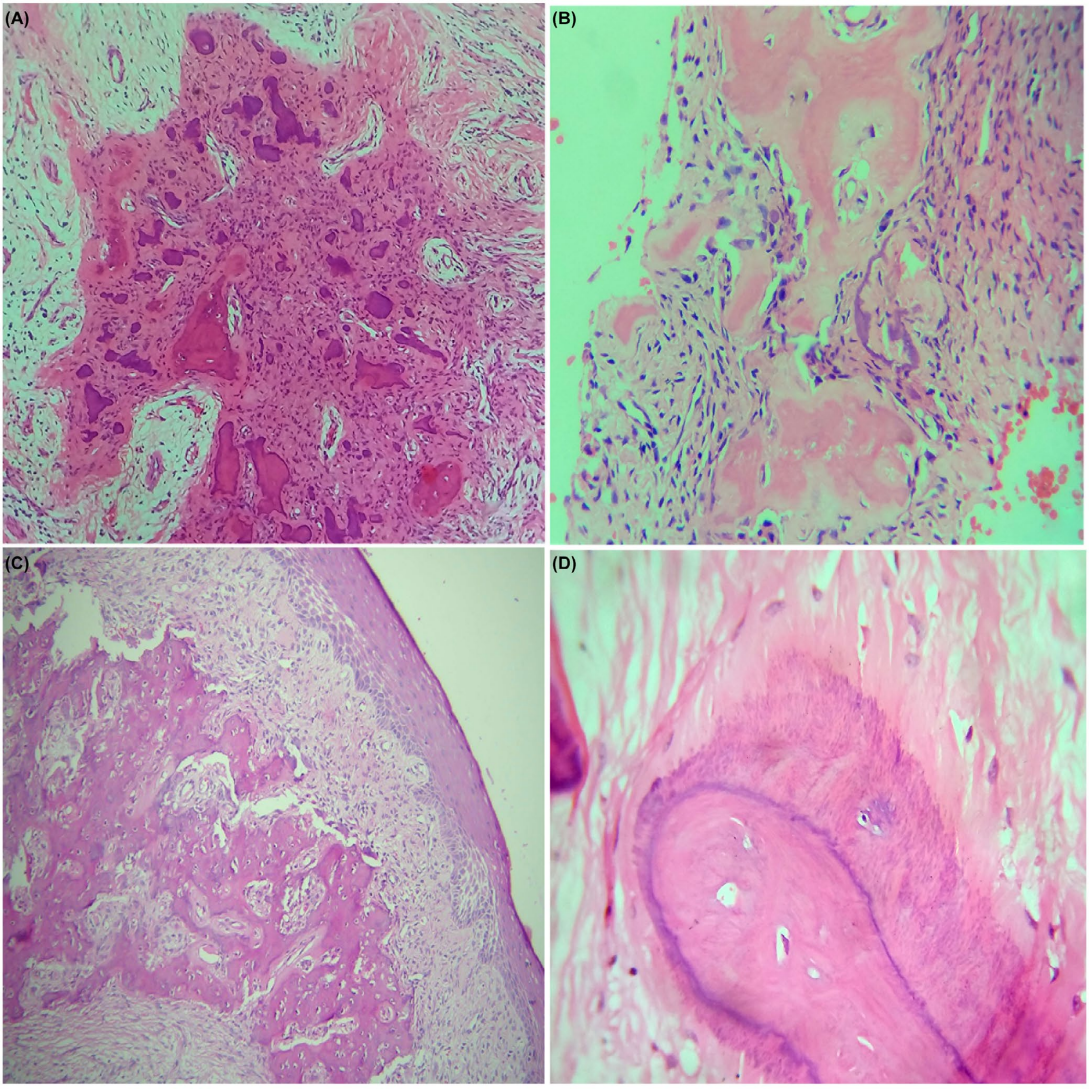
Histopathological features of peripheral ossifying fibroma exhibiting; woven bone and cementum‐like materials (A, 10×); hyalinized eosinophilic area in attempt of bone formation (B, 10×); extended bone formation approaching atrophic epithelium (C, 10×); brush border pattern (D, 40×; H&E)

All three patterns of calcification were evident in present review. Type I, that is, woven and lamellar type trabeculae, was a common finding in all 23 cases however was predominated by woven bone. Woven bone mainly presented as intense eosinophilic, irregular, short interconnected trabeculae with randomly distributed osteocytes and basophilic rim at the periphery of variable size (Figure 2A). Areas of hyalinization in attempt of bone formation were also evident (Figure 2B). Lamellar pattern of bone shows distinct rest line and reversal line with broad interconnecting trabeculae with or without osteoblastic rimming. One of the cases showed extensive trabeculae patterns which had approached near to the epithelium which was atrophic (Figure 2C).

**FIGURE 3:**
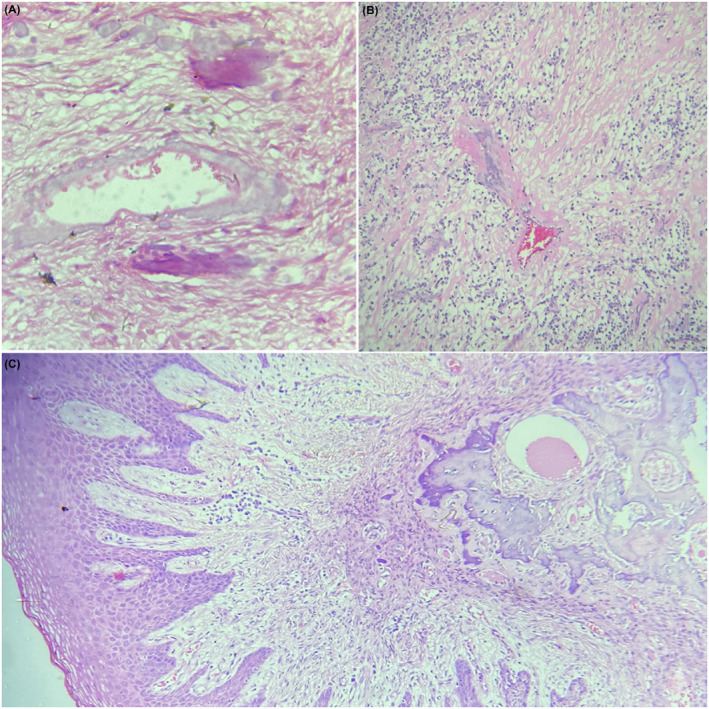
Diverse histopathological presentation with; perivascular calcification (A, 40×) and hyalinization (B, 10×); Three distinct zones from epithelium till deeper layers of connective tissue in peripheral ossifying fibroma (C, 4×; H&E)

Cementum‐like globules and dystrophic calcification were always seen along with trabeculae pattern. Few of the cases showed brush border pattern in the cementum‐like calcification (Figure 2D). Two of the cases showed dystrophic type calcification along and adjacent to the wall of blood vessels (Figure 3A). A case with hyalinization around the forming blood vessel was also observed (Figure 3B).

Irrespective of detail histopathological variations, a comprehensive evaluation of microscopic presentation for cases of POF showed three identifiable layers from surface epithelium to deep connective tissue. The first being the surface epithelial layer, second being the fibro‐cellular layer with vasculature, inflammatory cells, and active fibroblast, and the third deep layer with bone or calcification along with adjacent proliferating fibroblast (Figure 3C).

## 3 DISCUSSION

Peripheral ossifying fibroma is a focal, slow growing, reactive lesion arising from pluripotent cells of periodontal ligament.^1^, ^2^, ^3^ The lesion is synonymously known as peripheral cementifying fibroma, calcifying or ossifying fibroid epulis, and peripheral fibroma with calcification.^9^ The lesion might be sessile or pedunculated, predominately associated with anterior maxilla mainly in the second decade of life with female predilection. Radiographically it seldom shows any bony change except pressure associated cupping defect, occasional displacement of tooth, and diffuse radiopaque calcification areas. However, in large lesion of long duration might show destructive changes of the bone.^3^, ^10^, ^11^ Histopathologically POF is non‐capsulated which shows lining of stratified squamous epithelium with haphazardly scattered calcified areas in the background of highly cellular connective tissue.^12^

The study conducted by Buchner et al in 1987,^6^ on histopathological aspect considering 207 cases of POF was one of the most extensive descriptive analysis made for this pathology till date. The foremost concern of the analysis made was the summary of three distinct zones within the epithelium and connective tissue and categorization of all the probable types of calcification. The descriptions for the identification of these calcifications were so comprehensive that it is adapted by many authors till date not only in POF rather in many pathologies included within the umbrella of reactive lesion of oral cavity.

The three zones identified were as follows:

Zone I: The superficial ulcerated zone covered with fibrinous exudate with enmeshed polymorphonuclear neutrophil and debris. Adjacent epithelium is of parakeratinized and often hyperplastic.

Zone II: The zone beneath surface ulceration composed of proliferating fibroblast with dystrophic and globular pattern of calcification. Varied presentation for collagen, vascularity, and inflammatory cells.

Zone III: The deepest zone with more collagen and less vascular connective tissue. Chronic inflammatory cells ranging from few to absent. Spindle‐shaped fibroblast with fasciculate arrangement.

Epithelial ulceration is a common presentation of POF.^1^, ^8^, ^10^ Buchner et al correlated ulcerated epithelium to be prevalent in cases with dystrophic calcification. The study summarized ulcerated and non‐ulcerated epithelium was a common spectrum of single lesion which initiate with ulceration, increased activities of fibroblast with dystrophic calcification and osteogenesis. The same lesion than heals followed by maturation of the hard tissue deposits.^6^

Calcification, one of the characteristic finding of POF is supposed to be initiated around the wall of blood vessels.^6^ POF shows different form of calcification with variation in its quantity and pattern of distribution. The calcified hard tissue is supposed either as bone or cementum and is justified by, POF originating from mesenchymal cells of periodontal ligament. The pluripotent cells of the periodontal ligament have the seeming capability to metaplastically change into osteoblasts, cementoblasts, or fibroblasts, in response to irritants such as calculus, bacterial plaque, orthodontic appliances, ill‐adapted crowns, and irregular restorations, resulting into unique inflammatory hyperplasia, the peripheral ossifying fibroma.^1^, ^2^, ^3^, ^6^, ^9^

Pyogenic granuloma is the most common differential diagnosis with closest resemblance to POF both clinically and histopathologically. The initial stage of POF characterized by ulcerated epithelium, active fibroblast, and few dystrophic type calcification simulated to pyogenic granuloma.^2^, ^3^, ^6^, ^11^ Peripheral ossifying fibroma is often considered to be lesion with high cellularity and less vascularity in comparison with pyogenic granuloma.^10^ However, this feature might not always stand to differentiate these two pathologies. Calcification is an inherent property of cells of periodontal ligament which is hypothesized as origin of POF. This point could be a concluding remark while dealing with the cases showing overlapping features of pyogenic granuloma and POF.^6^ Apart from pyogenic granuloma, there is a need of histopathological differentiation of POF from peripheral giant cell granuloma (PGCG) and peripheral odontogenic fibroma as well. In contrast to POF, PGCG shows distribution of multinucleated giant cells while peripheral odontogenic fibroma shows dispersed odontogenic epithelium within the stroma.^9^

Reactive and non‐neoplastic nature of POF^3^ makes its management suitable for local surgical excision with maintenance of oral hygiene. However, to avoid its high reoccurrence rate, extensive excision till bone or subjacent to the periosteum has been practiced.^1^, ^10^

## 4 CONCLUSION

Peripheral ossifying fibroma is a slow‐growing lesion with limited growth potential characterized by metaplasia of the connective tissue leading to production of bone, cementum, or dystrophic calcification. Uniform clinical presentations with occurrence among both males and females and among wide age range with varying histopathological features have increased the need to meticulously review the differential diagnoses before formulating a definite histopathological diagnosis, considering the recurrent nature of the pathology.

## CONFLICT OF INTEREST

None.

## AUTHOR CONTRIBUTIONS

All authors contributed to the design of this manuscript. AS, SK, NJ, and TR prepared the first draft. AS, TR, SLS, and MRJ prepared the final manuscript. SLS and MRJ: involved in direct patient management of the cases. AS, SK, and NJ: pathologists involved in the diagnosis of the cases.

## CONSENT

Written informed consent was obtained from the patient for publication of this case report and accompanying images.

## Data Availability

Data are available from the corresponding author on request.
